# Supplementary material for: Vertebrate-Aedes aegypti and Culex quinquefasciatus (Diptera)-arbovirus transmission networks: Non-human feeding revealed by meta-barcoding and next-generation sequencing
Source: PLoS Negl Trop Dis. 2020 Dec 31;14(12):e0008867. doi: 10.1371/journal.pntd.0008867 (PMC7806141; doi:10.1371/journal.pntd.0008867)
Supplement: S1 Table — The sequences of the M13F and M13R tails are in bold, while the COI-specific sequences are in regular font. The M13 tails served as second round PCR primer binding sites, on which Ion Torrent sequencing adapters and UMIs (IonXpress 1–96) were fused. (DOCX) [file pntd.0008867.s001.docx]

**S1_Table**: Vertebrate-specific primers used for first round PCR. The sequences of the M13F and M13R tails are in bold, while the COI-specific sequences are in regular font. The M13 tails served as second round PCR primer binding sites, on which Ion Torrent sequencing adapters and UMIs (IonXpress 1-96) were fused.

| **Primer Name** | **Sequence (5’->3’)** | **Direction** | **Reference** |
| --- | --- | --- | --- |
| BloodmealF1_t1 | **TGTAAAACGACGGCCAGT**ACCACWATTATTAAYATAAARCCMC | Forward | González-Mikel *et al*. [[1](#_ENREF_1)] |
| BloodmealF2_t1 | **TGTAAAACGACGGCCAGT**ACTACAGCAATTAACATAAAACCMC | Forward | González-Mikel *et al*. [[1](#_ENREF_1)] |
| VR1_t1 | **CAGGAAACAGCTATGAC**TAGACTTCTGGGTGGCCAAAGAATCA | Forward | Hernández-Triana *et al*. [[2](#_ENREF_2)] |
| VR1d_t1 | **CAGGAAACAGCTATGAC**TAGACTTCTGGGTGGCCRAARAAYCA | Reverse | Hernández-Triana *et al*.[[2](#_ENREF_2)] |
| VR1i_t1 | **CAGGAAACAGCTATGAC**TAGACTTCTGGGTGICCIAAIAAICA | Reverse | Hernández-Triana *et al*. [[2](#_ENREF_2)] |

**Reference**:

1. González MA, Prosser SW, Hernández-Triana LM, Alarcón-Elbal PM, Goiri F, López S, et al. Avian Feeding Preferences of Culex pipiens and Culiseta spp. Along an Urban-to-Wild Gradient in Northern Spain. Frontiers in Ecology and Evolution. 2020;8(352). doi: 10.3389/fevo.2020.568835.

2. Hernandez-Triana LM, Brugman VA, Prosser SWJ, Weland C, Nikolova N, Thorne L, et al. Molecular approaches for blood meal analysis and species identification of mosquitoes (Insecta: Diptera: Culicidae) in rural locations in southern England, United Kingdom. Zootaxa. 2017;4250(1):67-76. doi: 10.11646/zootaxa.4250.1.5. PubMed PMID: 28610033.
