# Supplementary material for: Vertebrate-Aedes aegypti and Culex quinquefasciatus (Diptera)-arbovirus transmission networks: Non-human feeding revealed by meta-barcoding and next-generation sequencing
Source: PLoS Negl Trop Dis. 2020 Dec 31;14(12):e0008867. doi: 10.1371/journal.pntd.0008867 (PMC7806141; doi:10.1371/journal.pntd.0008867)
Supplement: S2 Table — (DOCX) [file pntd.0008867.s002.docx]

**S2_Table**: Collection data of mosquitoes collected in Reynosa in May-June and September-November 2018 for this study.

| **Species** |  | **May-July** | **September-November** | **Total** |
| --- | --- | --- | --- | --- |
| *Aedes aegypti* | Male | 1,744 | 526 | 2270 |
|  | Female fed | 331 | 105 | 436 |
|  | Female unfed | 2030 | 475 | 2505 |
|  | Total caught | 4105 | 1106 | 5211 |
|  |  |  |  |  |
| *Culex quinquefasciatus* | Male | 6528 | 647 | 7175 |
|  | Female fed | 475 | 133 | 608 |
|  | Female unfed | 2653 | 275 | 2928 |
|  | Total caught | 9656 | 1055 | 10711 |
| **Overall** |  | **13,761** | **2,161** | **15,922** |
