# Supplementary material for: Vertebrate-Aedes aegypti and Culex quinquefasciatus (Diptera)-arbovirus transmission networks: Non-human feeding revealed by meta-barcoding and next-generation sequencing
Source: PLoS Negl Trop Dis. 2020 Dec 31;14(12):e0008867. doi: 10.1371/journal.pntd.0008867 (PMC7806141; doi:10.1371/journal.pntd.0008867)
Supplement: S3 Table — (DOCX) [file pntd.0008867.s003.docx]

**S3_Table**: Number of read counts for each host identification in individual *Aedes aegypti* and *Culex quinquefasciatus* examined through next-generation deep sequencing.

| **Sample ID** | **Mosquito identification** | **No. of reads** | **Host identification** |
| --- | --- | --- | --- |
| REYN012018A01 | *Aedes aegypti* | 7784 | *Homo sapiens* |
|  |  |  |  |
| REYN012018A02 | *Aedes aegypti* | 7964 | *Homo sapiens* |
|  |  |  |  |
| REYN012018A03 | *Aedes aegypti* | 5580 | *Homo sapiens* |
|  |  |  |  |
| REYN012018A04 | *Aedes aegypti* | 22995 | *Canis lupus* |
| REYN012018A05 | *Aedes aegypti* | 16027 | *Canis lupus* |
| REYN012018A05 | *Aedes aegypti* | 788 | *Homo sapiens* |
| REYN012018A06 | *Aedes aegypti* | 15706 | *Canis lupus* |
| REYN012018A06 | *Aedes aegypti* | 1632 | *Homo sapiens* |
| REYN012018A07 | *Aedes aegypti* | 25297 | *Canis lupus* |
| REYN012018A08 | *Aedes aegypti* | 5785 | *Homo sapiens* |
|  |  |  |  |
| REYN012018A09 | *Aedes aegypti* | 24994 | *Canis lupus* |
| REYN012018A10 | *Aedes aegypti* | 18463 | *Canis lupus* |
| REYN012018A10 | *Aedes aegypti* | 4742 | *Homo sapiens* |
| REYN012018A11 | *Aedes aegypti* | 2876 | *Homo sapiens* |
| REYN012018A11 | *Aedes aegypti* | 153 | *Canis lupus* |
| REYN012018A12 | *Aedes aegypti* | 20277 | *Canis lupus* |
| REYN012018A12 | *Aedes aegypti* | 8560 | *Homo sapiens* |
| REYN012018B01 | *Aedes aegypti* | 15587 | *Homo sapiens* |
| REYN012018B02 | *Aedes aegypti* | 11713 | *Equus ferus caballus* |
| REYN012018B02 | *Aedes aegypti* | 10751 | *Homo sapiens* |
| REYN012018B03 | *Aedes aegypti* | 16619 | *Homo sapiens* |
| REYN012018B04 | *Aedes aegypti* | 9706 | *Homo sapiens* |
|  |  |  |  |
| REYN012018B05 | *Aedes aegypti* | 3559 | *Homo sapiens* |
|  |  |  |  |
| REYN012018B06 | *Aedes aegypti* | 4796 | *Felis silvestris silvestris* |
| REYN012018B07 | *Aedes aegypti* | 16540 | *Homo sapiens* |
| REYN012018B08 | *Aedes aegypti* | 20673 | *Homo sapiens* |
| REYN012018B09 | *Aedes aegypti* | 24181 | *Homo sapiens* |
| REYN012018B09 | *Aedes aegypti* | 115 | *Canis lupus* |
| REYN012018B10 | *Aedes aegypti* | 4768 | *Homo sapiens* |
| REYN012018B12 | *Aedes aegypti* | 8497 | *Homo sapiens* |
|  |  |  |  |
| REYN012018C02 | *Aedes aegypti* | 7053 | *Homo sapiens* |
|  |  |  |  |
| REYN012018C03 | *Aedes aegypti* | 16008 | *Canis lupus* |
| REYN012018C04 | *Aedes aegypti* | 10395 | *Felis silvestris silvestris* |
| REYN012018C05 | *Aedes aegypti* | 24083 | *Felis silvestris silvestris* |
| REYN012018C05 | *Aedes aegypti* | 138 | *Canis lupus* |
| REYN012018C06 | *Aedes aegypti* | 10571 | *Felis silvestris silvestris* |
| REYN012018C07 | *Aedes aegypti* | 6743 | *Felis silvestris silvestris* |
| REYN012018C08 | *Aedes aegypti* | 12106 | *Homo sapiens* |
| REYN012018C09 | *Aedes aegypti* | 18332 | *Felis silvestris silvestris* |
| REYN012018C10 | *Aedes aegypti* | 22361 | *Felis silvestris silvestris* |
| REYN012018C11 | *Aedes aegypti* | 17330 | *Canis lupus* |
| REYN012018C12 | *Aedes aegypti* | 14339 | *Felis silvestris silvestris* |
| REYN012018D01 | *Aedes aegypti* | 11489 | *Felis silvestris silvestris* |
| REYN012018D02 | *Aedes aegypti* | 22919 | *Felis silvestris silvestris* |
| REYN012018D02 | *Aedes aegypti* | 4218 | *Homo sapiens* |
| REYN012018D03 | *Aedes aegypti* | 21425 | *Canis lupus* |
| REYN012018D03 | *Aedes aegypti* | 3727 | *Homo sapiens* |
| REYN012018D04 | *Aedes aegypti* | 760 | *Felis silvestris silvestris* |
| REYN012018D05 | *Aedes aegypti* | 5076 | *Felis silvestris silvestris* |
| REYN012018D07 | *Aedes aegypti* | 8017 | *Canis lupus* |
| REYN012018D08 | *Aedes aegypti* | 3336 | *Felis silvestris silvestris* |
| REYN012018D09 | *Aedes aegypti* | 1039 | *Felis silvestris silvestris* |
| REYN012018D10 | *Aedes aegypti* | 25701 | *Felis silvestris silvestris* |
| REYN012018D11 | *Aedes aegypti* | 9453 | *Homo sapiens* |
| REYN012018D11 | *Aedes aegypti* | 146 | *Canis lupus* |
| REYN012018D12 | *Aedes aegypti* | 27880 | *Homo sapiens* |
| REYN012018E01 | *Aedes aegypti* | 276 | *Canis lupus* |
| REYN012018E02 | *Aedes aegypti* | 3256 | *Canis lupus* |
| REYN012018E06 | *Aedes aegypti* | 13105 | *Homo sapiens* |
| REYN012018E07 | *Aedes aegypti* | 21606 | *Canis lupus* |
| REYN012018E08 | *Aedes aegypti* | 25673 | *Canis lupus* |
| REYN012018E09 | *Aedes aegypti* | 14328 | *Canis lupus* |
| REYN012018E09 | *Aedes aegypti* | 648 | *Cairina moschata* |
| REYN012018E10 | *Aedes aegypti* | 19173 | *Homo sapiens* |
| REYN012018E11 | *Aedes aegypti* | 12656 | *Canis lupus* |
| REYN012018E12 | *Aedes aegypti* | 22779 | *Homo sapiens* |
| REYN012018E12 | *Aedes aegypti* | 172 | *Canis lupus* |
| REYN012018F01 | *Aedes aegypti* | 26674 | *Canis lupus* |
| REYN012018F01 | *Aedes aegypti* | 273 | *Bos taurus* |
| REYN012018F02 | *Aedes aegypti* | 20630 | *Canis lupus* |
| REYN012018F03 | *Aedes aegypti* | 22226 | *Canis lupus* |
| REYN012018F04 | *Aedes aegypti* | 22843 | *Canis lupus* |
| REYN012018F06 | *Aedes aegypti* | 18508 | *Canis lupus* |
| REYN012018F07 | *Aedes aegypti* | 20492 | *Canis lupus* |
| REYN012018F08 | *Aedes aegypti* | 15114 | *Homo sapiens* |
| REYN012018F09 | *Aedes aegypti* | 33275 | *Canis lupus* |
| REYN012018F10 | *Aedes aegypti* | 9907 | *Canis lupus* |
| REYN012018F11 | *Aedes aegypti* | 16823 | *Homo sapiens* |
|  |  |  |  |
| REYN012018G01 | *Aedes aegypti* | 17494 | *Homo sapiens* |
| REYN012018G01 | *Aedes aegypti* | 136 | *Canis lupus* |
| REYN012018G03 | *Aedes aegypti* | 23481 | *Canis lupus* |
| REYN012018G03 | *Aedes aegypti* | 121 | *Gallus gallus* |
| REYN012018G04 | *Aedes aegypti* | 15339 | *Homo sapiens* |
| REYN012018G04 | *Aedes aegypti* | 123 | *Canis lupus* |
| REYN012018G05 | *Aedes aegypti* | 17415 | *Canis lupus* |
| REYN012018G06 | *Aedes aegypti* | 11416 | *Canis lupus* |
| REYN012018G07 | *Aedes aegypti* | 10092 | *Trachemys scripta* |
| REYN012018G08 | *Aedes aegypti* | 25779 | *Canis lupus* |
| REYN012018G09 | *Aedes aegypti* | 19231 | *Canis lupus* |
| REYN012018G09 | *Aedes aegypti* | 8051 | *Homo sapiens* |
| REYN012018G10 | *Aedes aegypti* | 17338 | *Homo sapiens* |
| REYN012018G10 | *Aedes aegypti* | 11280 | *Canis lupus* |
| REYN012018G11 | *Aedes aegypti* | 3331 | *Homo sapiens* |
|  |  |  |  |
| REYN012018G12 | *Aedes aegypti* | 24227 | *Canis lupus* |
| REYN012018H01 | *Aedes aegypti* | 9161 | *Canis lupus* |
| REYN012018H01 | *Aedes aegypti* | 6739 | *Homo sapiens* |
| REYN012018H02 | *Aedes aegypti* | 19491 | *Canis lupus* |
| REYN012018H03 | *Aedes aegypti* | 10302 | *Canis lupus* |
| REYN012018H04 | *Aedes aegypti* | 10201 | *Canis lupus* |
| REYN012018H05 | *Aedes aegypti* | 15077 | *Homo sapiens* |
| REYN012018H06 | *Aedes aegypti* | 21854 | *Canis lupus* |
| REYN012018H09 | *Aedes aegypti* | 17963 | *Canis lupus* |
| REYN012018H11 | *Aedes aegypti* | 9420 | *Canis lupus* |
| REYN02A2018A01 | *Aedes aegypti* | 32133 | *Homo sapiens* |
| REYN02A2018A02 | *Aedes aegypti* | 29310 | *Felis silvestris silvestris* |
| REYN02A2018A03 | *Aedes aegypti* | 5983 | *Canis lupus* |
| REYN02A2018A04 | *Aedes aegypti* | 10129 | *Homo sapiens* |
| REYN02A2018A05 | *Aedes aegypti* | 28170 | *Canis lupus* |
| REYN02A2018A06 | *Aedes aegypti* | 3463 | *Canis lupus* |
| REYN02A2018A07 | *Aedes aegypti* | 8269 | *Canis lupus* |
| REYN02A2018A08 | *Aedes aegypti* | 7500 | *Canis lupus* |
| REYN02A2018A09 | *Aedes aegypti* | 3545 | *Felis silvestris silvestris* |
| REYN02A2018A10 | *Aedes aegypti* | 23635 | *Felis silvestris silvestris* |
| REYN02A2018A10 | *Aedes aegypti* | 127 | *Canis lupus* |
| REYN02A2018A11 | *Aedes aegypti* | 477 | *Felis silvestris silvestris* |
| REYN02A2018A11 | *Aedes aegypti* | 140 | *Canis lupus* |
| REYN02A2018A12 | *Aedes aegypti* | 22382 | *Homo sapiens* |
| REYN02A2018A12 | *Aedes aegypti* | 113 | *Canis lupus* |
| REYN02A2018B01 | *Aedes aegypti* | 20411 | *Canis lupus* |
| REYN02A2018B02 | *Aedes aegypti* | 28426 | *Canis lupus* |
| REYN02A2018B03 | *Aedes aegypti* | 6842 | *Homo sapiens* |
|  |  |  |  |
| REYN02A2018B04 | *Aedes aegypti* | 18552 | *Homo sapiens* |
| REYN02A2018B05 | *Aedes aegypti* | 34614 | *Homo sapiens* |
| REYN02A2018B05 | *Aedes aegypti* | 127 | *Canis lupus* |
| REYN02A2018B06 | *Aedes aegypti* | 27741 | *Canis lupus* |
| REYN02A2018B07 | *Aedes aegypti* | 22148 | *Homo sapiens* |
| REYN02A2018B07 | *Aedes aegypti* | 124 | *Canis lupus* |
| REYN02A2018B08 | *Aedes aegypti* | 24382 | *Canis lupus* |
| REYN02A2018B09 | *Aedes aegypti* | 28510 | *Homo sapiens* |
| REYN02A2018B09 | *Aedes aegypti* | 142 | *Canis lupus* |
| REYN02A2018B10 | *Aedes aegypti* | 26406 | *Canis lupus* |
| REYN02A2018B11 | *Aedes aegypti* | 25241 | *Canis lupus* |
| REYN02A2018B12 | *Aedes aegypti* | 16632 | *Canis lupus* |
| REYN02A2018C01 | *Aedes aegypti* | 8875 | *Canis lupus* |
| REYN02A2018C02 | *Aedes aegypti* | 27665 | *Canis lupus* |
| REYN02A2018C03 | *Aedes aegypti* | 19842 | *Canis lupus* |
| REYN02A2018C04 | *Aedes aegypti* | 23467 | *Homo sapiens* |
| REYN02A2018C05 | *Aedes aegypti* | 26639 | *Felis silvestris silvestris* |
| REYN02A2018C06 | *Aedes aegypti* | 20567 | *Canis lupus* |
| REYN02A2018C07 | *Aedes aegypti* | 19411 | *Felis silvestris silvestris* |
| REYN02A2018C08 | *Aedes aegypti* | 28603 | *Canis lupus* |
| REYN02A2018C09 | *Aedes aegypti* | 6395 | *Homo sapiens* |
|  |  |  |  |
| REYN02A2018C10 | *Aedes aegypti* | 29603 | *Canis lupus* |
| REYN02A2018C11 | *Aedes aegypti* | 27088 | *Canis lupus* |
| REYN02A2018C12 | *Aedes aegypti* | 12516 | *Canis lupus* |
| REYN02A2018D01 | *Aedes aegypti* | 7232 | *Homo sapiens* |
|  |  |  |  |
| REYN02A2018D01 | *Aedes aegypti* | 134 | *Canis lupus* |
| REYN02A2018D02 | *Aedes aegypti* | 6904 | *Felis silvestris silvestris* |
| REYN02A2018D02 | *Aedes aegypti* | 146 | *Canis lupus* |
| REYN02A2018D04 | *Aedes aegypti* | 19971 | *Canis lupus* |
| REYN02A2018D05 | *Aedes aegypti* | 8902 | *Homo sapiens* |
| REYN02A2018D06 | *Aedes aegypti* | 10500 | *Canis lupus* |
| REYN02A2018D07 | *Aedes aegypti* | 395 | *Canis lupus* |
| REYN02A2018D08 | *Aedes aegypti* | 461 | *Bos taurus* |
| REYN02A2018D08 | *Aedes aegypti* | 105 | *Canis lupus* |
| REYN02A2018D10 | *Aedes aegypti* | 26183 | *Canis lupus* |
| REYN02A2018D11 | *Aedes aegypti* | 19434 | *Homo sapiens* |
| REYN02A2018D12 | *Aedes aegypti* | 682 | *Felis silvestris silvestris* |
| REYN02A2018E01 | *Aedes aegypti* | 128 | *Homo sapiens* |
| REYN02A2018E02 | *Aedes aegypti* | 7848 | *Homo sapiens* |
| REYN02A2018E03 | *Aedes aegypti* | 152 | *Canis lupus* |
| REYN02A2018E03 | *Aedes aegypti* | 128 | *Homo sapiens* |
| REYN02A2018E04 | *Aedes aegypti* | 23533 | *Canis lupus* |
| REYN02A2018E05 | *Aedes aegypti* | 20817 | *Homo sapiens* |
| REYN02A2018E05 | *Aedes aegypti* | 128 | *Canis lupus* |
| REYN02A2018E06 | *Aedes aegypti* | 20783 | *Felis silvestris silvestris* |
| REYN02A2018E06 | *Aedes aegypti* | 3166 | *Canis lupus* |
| REYN02A2018E07 | *Aedes aegypti* | 22387 | *Homo sapiens* |
| REYN02A2018E08 | *Aedes aegypti* | 2726 | *Homo sapiens* |
| REYN02A2018E08 | *Aedes aegypti* | 2154 | *Canis lupus* |
|  |  |  |  |
| REYN02A2018E09 | *Aedes aegypti* | 672 | *Canis lupus* |
| REYN02A2018E09 | *Aedes aegypti* | 563 | *Felis silvestris silvestris* |
| REYN02A2018E10 | *Aedes aegypti* | 26903 | *Canis lupus* |
| REYN02A2018E11 | *Aedes aegypti* | 28313 | *Homo sapiens* |
| REYN02A2018E11 | *Aedes aegypti* | 107 | *Canis lupus* |
| REYN02A2018E12 | *Aedes aegypti* | 11995 | *Canis lupus* |
| REYN02A2018E12 | *Aedes aegypti* | 9668 | *Homo sapiens* |
| REYN02A2018F02 | *Aedes aegypti* | 31083 | *Canis lupus* |
| REYN02A2018F03 | *Aedes aegypti* | 8652 | *Canis lupus* |
| REYN02A2018F04 | *Aedes aegypti* | 14213 | *Canis lupus* |
| REYN02A2018F05 | *Aedes aegypti* | 7897 | *Homo sapiens* |
|  |  |  |  |
| REYN02A2018F06 | *Aedes aegypti* | 4816 | *Homo sapiens* |
|  |  |  |  |
| REYN02A2018F07 | *Aedes aegypti* | 8925 | *Homo sapiens* |
|  |  |  |  |
| REYN02A2018F07 | *Aedes aegypti* | 161 | *Canis lupus* |
| REYN02A2018F08 | *Aedes aegypti* | 28276 | *Felis silvestris silvestris* |
| REYN02A2018F08 | *Aedes aegypti* | 152 | *Canis lupus* |
| REYN02A2018F10 | *Aedes aegypti* | 11786 | *Felis silvestris silvestris* |
| REYN02A2018F11 | *Aedes aegypti* | 18935 | *Canis lupus* |
| REYN02A2018F12 | *Aedes aegypti* | 17062 | *Canis lupus* |
| REYN02A2018G01 | *Aedes aegypti* | 9782 | *Homo sapiens* |
|  |  |  |  |
| REYN02A2018G02 | *Aedes aegypti* | 724 | *Canis lupus* |
| REYN032018A01 | *Culex quinquefasciatus* | 214 | *Canis lupus* |
| REYN032018A04 | *Culex quinquefasciatus* | 30785 | *Canis lupus* |
| REYN032018A05 | *Culex quinquefasciatus* | 18931 | *Streptopelia decaocto* |
| REYN032018A06 | *Culex quinquefasciatus* | 19166 | *Canis lupus* |
| REYN032018A07 | *Culex quinquefasciatus* | 36450 | *Canis lupus* |
| REYN032018A08 | *Culex quinquefasciatus* | 5461 | *Homo sapiens* |
| REYN032018A09 | *Culex quinquefasciatus* | 17599 | *Canis lupus* |
| REYN032018A09 | *Culex quinquefasciatus* | 6376 | *Columba livia* |
| REYN032018A10 | *Culex quinquefasciatus* | 371 | *Canis lupus* |
| REYN032018A11 | *Culex quinquefasciatus* | 21692 | *Canis lupus* |
| REYN032018A12 | *Culex quinquefasciatus* | 30842 | *Gallus sp.* |
|  |  |  |  |
| REYN032018A12 | *Culex quinquefasciatus* | 153 | *Canis lupus* |
| REYN032018B01 | *Culex quinquefasciatus* | 911 | *Zenaida macroura* |
| REYN032018B01 | *Culex quinquefasciatus* | 108 | *Canis lupus* |
| REYN032018B02 | *Culex quinquefasciatus* | 29140 | *Didelphis virginiana* |
| REYN032018B03 | *Culex quinquefasciatus* | 27430 | *Passer domesticus* |
| REYN032018B04 | *Culex quinquefasciatus* | 9721 | *Canis lupus* |
| REYN032018B04 | *Culex quinquefasciatus* | 3682 | *Passer domesticus* |
| REYN032018B05 | *Culex quinquefasciatus* | 31074 | *Canis lupus* |
| REYN032018B06 | *Culex quinquefasciatus* | 20587 | *Felis silvestris silvestris* |
| REYN032018B07 | *Culex quinquefasciatus* | 19927 | *Canis lupus* |
| REYN032018B08 | *Culex quinquefasciatus* | 16236 | *Canis lupus* |
| REYN032018B08 | *Culex quinquefasciatus* | 344 | *Passer domesticus* |
| REYN032018B09 | *Culex quinquefasciatus* | 26328 | *Canis lupus* |
| REYN032018B11 | *Culex quinquefasciatus* | 25795 | *Gallus gallus* |
| REYN032018B12 | *Culex quinquefasciatus* | 23185 | *Lasiurus intermedius* |
| REYN032018C01 | *Culex quinquefasciatus* | 24211 | *Canis lupus* |
| REYN032018C02 | *Culex quinquefasciatus* | 19089 | *Zenaida macroura* |
| REYN032018C03 | *Culex quinquefasciatus* | 17023 | *Canis lupus* |
| REYN032018C04 | *Culex quinquefasciatus* | 26754 | *Zenaida macroura* |
| REYN032018C05 | *Culex quinquefasciatus* | 36032 | *Canis lupus* |
| REYN032018C06 | *Culex quinquefasciatus* | 814 | *Canis lupus* |
| REYN032018C07 | *Culex quinquefasciatus* | 22285 | *Canis lupus* |
| REYN032018C09 | *Culex quinquefasciatus* | 7708 | *Didelphis virginiana* |
| REYN032018C11 | *Culex quinquefasciatus* | 268 | *Canis lupus* |
| REYN032018C12 | *Culex quinquefasciatus* | 20574 | *Canis lupus* |
| REYN032018D01 | *Culex quinquefasciatus* | 23205 | *Canis lupus* |
| REYN032018D02 | *Culex quinquefasciatus* | 617 | *Canis lupus* |
| REYN032018D04 | *Culex quinquefasciatus* | 139 | *Gallus gallus* |
| REYN032018D05 | *Culex quinquefasciatus* | 28133 | *Canis lupus* |
| REYN032018D06 | *Culex quinquefasciatus* | 22219 | *Canis lupus* |
| REYN032018D07 | *Culex quinquefasciatus* | 15379 | *Canis lupus* |
| REYN032018D08 | *Culex quinquefasciatus* | 20807 | *Canis lupus* |
| REYN032018D09 | *Culex quinquefasciatus* | 30824 | *Columbina inca* |
| REYN032018D10 | *Culex quinquefasciatus* | 19749 | *Canis lupus* |
| REYN032018D11 | *Culex quinquefasciatus* | 19942 | *Canis lupus* |
| REYN032018D11 | *Culex quinquefasciatus* | 8026 | *Felis silvestris silvestris* |
| REYN032018D12 | *Culex quinquefasciatus* | 38125 | *Passer domesticus* |
| REYN032018E01 | *Culex quinquefasciatus* | 17507 | *Gopherus berlandieri* |
| REYN032018E01 | *Culex quinquefasciatus* | 937 | *Didelphis virginiana* |
| REYN032018E02 | *Culex quinquefasciatus* | 23813 | *Canis lupus* |
| REYN032018E04 | *Culex quinquefasciatus* | 16262 | *Canis lupus* |
| REYN032018E05 | *Culex quinquefasciatus* | 134 | *Canis lupus* |
| REYN032018E06 | *Culex quinquefasciatus* | 25404 | *Canis lupus* |
| REYN032018E07 | *Culex quinquefasciatus* | 21534 | *Gallus gallus* |
| REYN032018E08 | *Culex quinquefasciatus* | 18938 | *Canis lupus* |
| REYN032018E10 | *Culex quinquefasciatus* | 19983 | *Zenaida macroura* |
| REYN032018E10 | *Culex quinquefasciatus* | 131 | *Canis lupus* |
| REYN032018E11 | *Culex quinquefasciatus* | 14723 | *Gallus gallus* |
| REYN032018E11 | *Culex quinquefasciatus* | 120 | *Canis lupus* |
| REYN032018E12 | *Culex quinquefasciatus* | 28119 | *Canis lupus* |
| REYN032018F01 | *Culex quinquefasciatus* | 6842 | *Canis lupus* |
| REYN032018F03 | *Culex quinquefasciatus* | 29149 | *Canis lupus* |
| REYN032018F04 | *Culex quinquefasciatus* | 23762 | *Canis lupus* |
| REYN032018F05 | *Culex quinquefasciatus* | 7754 | *Canis lupus* |
| REYN032018F05 | *Culex quinquefasciatus* | 4542 | *Bos taurus* |
| REYN032018F06 | *Culex quinquefasciatus* | 18822 | *Streptopelia decaocto* |
| REYN032018F07 | *Culex quinquefasciatus* | 15728 | *Streptopelia decaocto* |
| REYN032018F07 | *Culex quinquefasciatus* | 9379 | *Canis lupus* |
| REYN032018F08 | *Culex quinquefasciatus* | 28737 | *Columbina inca* |
| REYN032018F09 | *Culex quinquefasciatus* | 24894 | *Columbina inca* |
| REYN032018F09 | *Culex quinquefasciatus* | 22522 | *Canis lupus* |
| REYN032018F10 | *Culex quinquefasciatus* | 19041 | *Canis lupus* |
| REYN032018F10 | *Culex quinquefasciatus* | 3129 | *Columbina inca* |
| REYN032018F11 | *Culex quinquefasciatus* | 24548 | *Canis lupus* |
| REYN032018F12 | *Culex quinquefasciatus* | 4911 | *Canis lupus* |
| REYN032018F12 | *Culex quinquefasciatus* | 3054 | *Streptopelia decaocto* |
| REYN032018G01 | *Culex quinquefasciatus* | 15034 | *Canis lupus* |
| REYN032018G02 | *Culex quinquefasciatus* | 22567 | *Homo sapiens* |
| REYN032018G02 | *Culex quinquefasciatus* | 293 | *Canis lupus* |
| REYN032018G03 | *Culex quinquefasciatus* | 188 | *Canis lupus* |
| REYN032018G05 | *Culex quinquefasciatus* | 21302 | *Canis lupus* |
| REYN032018G06 | *Culex quinquefasciatus* | 12764 | *Streptopelia decaocto* |
| REYN032018G07 | *Culex quinquefasciatus* | 166 | *Canis lupus* |
| REYN032018G10 | *Culex quinquefasciatus* | 17469 | *Canis lupus* |
| REYN032018G11 | *Culex quinquefasciatus* | 19451 | *Gallus gallus* |
| REYN032018G12 | *Culex quinquefasciatus* | 20061 | *Zenaida macroura* |
| REYN032018G12 | *Culex quinquefasciatus* | 5407 | *Gallus sp.* |
|  |  |  |  |
| REYN032018H01 | *Culex quinquefasciatus* | 20182 | *Canis lupus* |
| REYN032018H02 | *Culex quinquefasciatus* | 25163 | *Canis lupus* |
| REYN032018H05 | *Culex quinquefasciatus* | 16984 | *Canis lupus* |
| REYN032018H06 | *Culex quinquefasciatus* | 6462 | *Canis lupus* |
| REYN032018H07 | *Culex quinquefasciatus* | 14936 | *Canis lupus* |
| REYN032018H08 | *Culex quinquefasciatus* | 17200 | *Canis lupus* |
| REYN032018H09 | *Culex quinquefasciatus* | 12051 | *Anas sp.* |
|  |  |  |  |
| REYN032018H11 | *Culex quinquefasciatus* | 16840 | *Canis lupus* |
| REYN032018H11 | *Culex quinquefasciatus* | 102 | *Anas platyrhynchos* |
| REYN042018A01 | *Culex quinquefasciatus* | 33311 | *Canis lupus* |
| REYN042018A02 | *Culex quinquefasciatus* | 12677 | *Canis lupus* |
| REYN042018A04 | *Culex quinquefasciatus* | 16332 | *Canis lupus* |
| REYN042018A05 | *Culex quinquefasciatus* | 13862 | *Canis lupus* |
| REYN042018A06 | *Culex quinquefasciatus* | 5634 | *Homo sapiens* |
| REYN042018A07 | *Culex quinquefasciatus* | 20917 | *Gallus sp.* |
|  |  |  |  |
| REYN042018A08 | *Culex quinquefasciatus* | 26008 | *Passer domesticus* |
| REYN042018A09 | *Culex quinquefasciatus* | 21053 | *Canis lupus* |
| REYN042018A10 | *Culex quinquefasciatus* | 29633 | *Canis lupus* |
| REYN042018A10 | *Culex quinquefasciatus* | 1147 | *Bos taurus* |
| REYN042018A11 | *Culex quinquefasciatus* | 24033 | *Streptopelia decaocto* |
| REYN042018A12 | *Culex quinquefasciatus* | 25516 | *Gallus gallus* |
| REYN042018B01 | *Culex quinquefasciatus* | 573 | *Canis lupus* |
| REYN042018B02 | *Culex quinquefasciatus* | 19858 | *Streptopelia decaocto* |
| REYN042018B03 | *Culex quinquefasciatus* | 25446 | *Canis lupus* |
| REYN042018B05 | *Culex quinquefasciatus* | 8373 | *Gallus gallus* |
| REYN042018B06 | *Culex quinquefasciatus* | 999 | *Streptopelia decaocto* |
| REYN042018B07 | *Culex quinquefasciatus* | 23573 | *Canis lupus* |
| REYN042018B08 | *Culex quinquefasciatus* | 5726 | *Homo sapiens* |
| REYN042018B08 | *Culex quinquefasciatus* | 556 | *Canis lupus* |
| REYN042018B09 | *Culex quinquefasciatus* | 18575 | *Canis lupus* |
| REYN042018B10 | *Culex quinquefasciatus* | 20688 | *Gallus gallus* |
| REYN042018B11 | *Culex quinquefasciatus* | 127 | *Gallus gallus* |
| REYN042018B12 | *Culex quinquefasciatus* | 22709 | *Gallus gallus* |
| REYN042018C01 | *Culex quinquefasciatus* | 29598 | *Canis lupus* |
| REYN042018C03 | *Culex quinquefasciatus* | 21530 | *Canis lupus* |
| REYN042018C04 | *Culex quinquefasciatus* | 10810 | *Canis lupus* |
| REYN042018C05 | *Culex quinquefasciatus* | 25852 | *Canis lupus* |
| REYN042018C06 | *Culex quinquefasciatus* | 12395 | *Canis lupus* |
| REYN042018C09 | *Culex quinquefasciatus* | 22962 | *Canis lupus* |
| REYN042018C10 | *Culex quinquefasciatus* | 32814 | *Homo sapiens* |
| REYN042018C12 | *Culex quinquefasciatus* | 19477 | *Canis lupus* |
| REYN042018D01 | *Culex quinquefasciatus* | 20998 | *Canis lupus* |
| REYN042018D02 | *Culex quinquefasciatus* | 21319 | *Streptopelia decaocto* |
| REYN042018D03 | *Culex quinquefasciatus* | 382 | *Didelphis virginiana* |
| REYN042018D04 | *Culex quinquefasciatus* | 16972 | *Canis lupus* |
| REYN042018D05 | *Culex quinquefasciatus* | 23974 | *Canis lupus* |
| REYN042018D06 | *Culex quinquefasciatus* | 28011 | *Canis lupus* |
| REYN042018D07 | *Culex quinquefasciatus* | 7364 | *Homo sapiens* |
| REYN042018D07 | *Culex quinquefasciatus* | 2879 | *Streptopelia decaocto* |
| REYN042018D08 | *Culex quinquefasciatus* | 16713 | *Canis lupus* |
| REYN042018D10 | *Culex quinquefasciatus* | 23782 | *Canis lupus* |
| REYN042018D11 | *Culex quinquefasciatus* | 29796 | *Canis lupus* |
| REYN042018D12 | *Culex quinquefasciatus* | 28341 | *Passer domesticus* |
| REYN042018E01 | *Culex quinquefasciatus* | 23704 | *Canis lupus* |
| REYN042018E02 | *Culex quinquefasciatus* | 12728 | *Didelphis sp.* |
|  |  |  |  |
| REYN042018E02 | *Culex quinquefasciatus* | 226 | *Canis lupus* |
| REYN042018E04 | *Culex quinquefasciatus* | 1093 | *Canis lupus* |
| REYN042018E05 | *Culex quinquefasciatus* | 14476 | *Canis lupus* |
| REYN042018E07 | *Culex quinquefasciatus* | 12422 | *Canis lupus* |
| REYN042018E07 | *Culex quinquefasciatus* | 1042 | *Homo sapiens* |
| REYN042018E08 | *Culex quinquefasciatus* | 25408 | *Canis lupus* |
| REYN042018E09 | *Culex quinquefasciatus* | 15687 | *Canis lupus* |
| REYN042018E10 | *Culex quinquefasciatus* | 17985 | *Canis lupus* |
| REYN042018E11 | *Culex quinquefasciatus* | 22013 | *Canis lupus* |
| REYN042018E12 | *Culex quinquefasciatus* | 11128 | *Canis lupus* |
| REYN042018F01 | *Culex quinquefasciatus* | 27848 | *Canis lupus* |
| REYN042018F02 | *Culex quinquefasciatus* | 29431 | *Canis lupus* |
| REYN042018F03 | *Culex quinquefasciatus* | 17304 | *Canis lupus* |
| REYN042018F04 | *Culex quinquefasciatus* | 31574 | *Canis lupus* |
| REYN042018F05 | *Culex quinquefasciatus* | 27536 | *Passer domesticus* |
| REYN042018F06 | *Culex quinquefasciatus* | 23496 | *Canis lupus* |
| REYN042018F07 | *Culex quinquefasciatus* | 18792 | *Canis lupus* |
| REYN042018F08 | *Culex quinquefasciatus* | 19919 | *Canis lupus* |
| REYN042018F09 | *Culex quinquefasciatus* | 337 | *Canis lupus* |
| REYN042018F10 | *Culex quinquefasciatus* | 12155 | *Felis silvestris silvestris* |
| REYN042018F11 | *Culex quinquefasciatus* | 116 | *Canis lupus* |
| REYN042018F12 | *Culex quinquefasciatus* | 25554 | *Canis lupus* |
| REYN042018G01 | *Culex quinquefasciatus* | 26884 | *Meleagris gallopavo* |
| REYN042018G01 | *Culex quinquefasciatus* | 3646 | *Gallus gallus* |
| REYN042018G03 | *Culex quinquefasciatus* | 27133 | *Canis lupus* |
| REYN042018G04 | *Culex quinquefasciatus* | 13198 | *Zenaida macroura* |
| REYN042018G05 | *Culex quinquefasciatus* | 23792 | *Canis lupus* |
| REYN042018G07 | *Culex quinquefasciatus* | 23546 | *Canis lupus* |
| REYN042018G08 | *Culex quinquefasciatus* | 5188 | *Canis lupus* |
| REYN042018G09 | *Culex quinquefasciatus* | 29552 | *Canis lupus* |
| REYN042018G10 | *Culex quinquefasciatus* | 33103 | *Canis lupus* |
| REYN042018G11 | *Culex quinquefasciatus* | 24780 | *Canis lupus* |
| REYN042018G12 | *Culex quinquefasciatus* | 104 | *Canis lupus* |
| REYN042018H01 | *Culex quinquefasciatus* | 11473 | *Canis lupus* |
| REYN042018H02 | *Culex quinquefasciatus* | 25301 | *Meleagris gallopavo* |
| REYN042018H03 | *Culex quinquefasciatus* | 25300 | *Zenaida macroura* |
| REYN042018H04 | *Culex quinquefasciatus* | 29060 | *Canis lupus* |
| REYN042018H05 | *Culex quinquefasciatus* | 17757 | *Zenaida macroura* |
| REYN042018H06 | *Culex quinquefasciatus* | 150 | *Meleagris gallopavo* |
| REYN042018H06 | *Culex quinquefasciatus* | 118 | *Canis lupus* |
| REYN042018H07 | *Culex quinquefasciatus* | 30263 | *Canis lupus* |
| REYN042018H07 | *Culex quinquefasciatus* | 6910 | *Meleagris gallopavo* |
| REYN042018H08 | *Culex quinquefasciatus* | 159 | *Canis lupus* |
| REYN042018H09 | *Culex quinquefasciatus* | 28810 | *Canis lupus* |
| REYN042018H10 | *Culex quinquefasciatus* | 27670 | *Canis lupus* |
| REYN042018H10 | *Culex quinquefasciatus* | 1333 | *Meleagris gallopavo* |
| REYN042018H11 | *Culex quinquefasciatus* | 38361 | *Canis lupus* |
| REYN052018A01 | *Culex quinquefasciatus* | 15141 | *Canis lupus* |
| REYN052018A01 | *Culex quinquefasciatus* | 236 | *Felis silvestris silvestris* |
| REYN052018A02 | *Culex quinquefasciatus* | 141 | *Canis lupus* |
| REYN052018A04 | *Culex quinquefasciatus* | 18177 | *Canis lupus* |
| REYN052018A04 | *Culex quinquefasciatus* | 228 | *Gallus gallus* |
| REYN052018A05 | *Culex quinquefasciatus* | 16815 | *Canis lupus* |
| REYN052018A06 | *Culex quinquefasciatus* | 15961 | *Canis lupus* |
| REYN052018A07 | *Culex quinquefasciatus* | 1527 | *Canis lupus* |
| REYN052018A08 | *Culex quinquefasciatus* | 25973 | *Gallus sp.* |
|  |  |  |  |
| REYN052018A09 | *Culex quinquefasciatus* | 10432 | *Passer domesticus* |
| REYN052018A09 | *Culex quinquefasciatus* | 7157 | *Canis lupus* |
| REYN052018A09 | *Culex quinquefasciatus* | 1874 | *Sus scrofa* |
| REYN052018A10 | *Culex quinquefasciatus* | 134 | *Canis lupus* |
| REYN052018A11 | *Culex quinquefasciatus* | 28742 | *Canis lupus* |
| REYN052018A12 | *Culex quinquefasciatus* | 5822 | *Felis silvestris silvestris* |
| REYN052018A12 | *Culex quinquefasciatus* | 132 | *Canis lupus* |
| REYN052018B03 | *Culex quinquefasciatus* | 11559 | *Canis lupus* |
| REYN052018B05 | *Culex quinquefasciatus* | 8575 | *Homo sapiens* |
|  |  |  |  |
| REYN052018B07 | *Culex quinquefasciatus* | 108 | *Canis lupus* |
| REYN052018B08 | *Culex quinquefasciatus* | 7466 | *Homo sapiens* |
| REYN052018B08 | *Culex quinquefasciatus* | 5913 | *Canis lupus* |
| REYN052018B09 | *Culex quinquefasciatus* | 21258 | *Canis lupus* |
| REYN052018B10 | *Culex quinquefasciatus* | 9638 | *Zenaida macroura* |
| REYN052018B11 | *Culex quinquefasciatus* | 8255 | *Homo sapiens* |
| REYN052018B12 | *Culex quinquefasciatus* | 11183 | *Homo sapiens* |
| REYN052018C03 | *Culex quinquefasciatus* | 115 | *Canis lupus* |
| REYN052018C04 | *Culex quinquefasciatus* | 12846 | *Canis lupus* |
| REYN052018C05 | *Culex quinquefasciatus* | 22917 | *Canis lupus* |
| REYN052018C06 | *Culex quinquefasciatus* | 17299 | *Gallus gallus* |
| REYN052018C08 | *Culex quinquefasciatus* | 21359 | *Canis lupus* |
| REYN052018C09 | *Culex quinquefasciatus* | 15558 | *Didelphis virginiana* |
| REYN052018C10 | *Culex quinquefasciatus* | 17233 | *Didelphis virginiana* |
| REYN052018C11 | *Culex quinquefasciatus* | 7652 | *Didelphis sp.* |
|  |  |  |  |
| REYN052018C11 | *Culex quinquefasciatus* | 103 | *Canis lupus* |
| REYN052018C12 | *Culex quinquefasciatus* | 294 | *Canis lupus* |
| REYN052018D01 | *Culex quinquefasciatus* | 16355 | *Gallus sp.* |
|  |  |  |  |
| REYN052018D02 | *Culex quinquefasciatus* | 13108 | *Canis lupus* |
| REYN052018D03 | *Culex quinquefasciatus* | 153 | *Passer domesticus* |
| REYN052018D03 | *Culex quinquefasciatus* | 100 | *Canis lupus* |
| REYN052018D04 | *Culex quinquefasciatus* | 26531 | *Gallus gallus* |
| REYN052018D05 | *Culex quinquefasciatus* | 12801 | *Canis lupus* |
| REYN052018D05 | *Culex quinquefasciatus* | 332 | *Sus scrofa* |
| REYN052018D06 | *Culex quinquefasciatus* | 158 | *Canis lupus* |
| REYN052018D07 | *Culex quinquefasciatus* | 27684 | *Canis lupus* |
| REYN052018D08 | *Culex quinquefasciatus* | 13939 | *Passer domesticus* |
| REYN052018D08 | *Culex quinquefasciatus* | 674 | *Canis lupus* |
| REYN052018D09 | *Culex quinquefasciatus* | 240 | *Canis lupus* |
| REYN052018D10 | *Culex quinquefasciatus* | 10995 | *Canis lupus* |
| REYN052018D11 | *Culex quinquefasciatus* | 20456 | *Canis lupus* |
| REYN052018D12 | *Culex quinquefasciatus* | 16584 | *Canis lupus* |
| REYN052018E01 | *Culex quinquefasciatus* | 11323 | *Canis lupus* |
| REYN052018E04 | *Culex quinquefasciatus* | 23183 | *Canis lupus* |
| REYN052018E05 | *Culex quinquefasciatus* | 22574 | *Canis lupus* |
| REYN052018E06 | *Culex quinquefasciatus* | 9637 | *Canis lupus* |
| REYN052018E07 | *Culex quinquefasciatus* | 10723 | *Canis lupus* |
| REYN052018E08 | *Culex quinquefasciatus* | 20125 | *Canis lupus* |
| REYN052018E10 | *Culex quinquefasciatus* | 10622 | *Anas platyrhynchos* |
| REYN052018E10 | *Culex quinquefasciatus* | 106 | *Canis lupus* |
| REYN052018E11 | *Culex quinquefasciatus* | 25316 | *Canis lupus* |
| REYN052018E12 | *Culex quinquefasciatus* | 23110 | *Canis lupus* |
| REYN052018E12 | *Culex quinquefasciatus* | 1068 | *Gallus gallus* |
| REYN052018F01 | *Culex quinquefasciatus* | 234 | *Canis lupus* |
| REYN052018F02 | *Culex quinquefasciatus* | 130 | *Canis lupus* |
| REYN052018F03 | *Culex quinquefasciatus* | 17483 | *Canis lupus* |
| REYN052018F04 | *Culex quinquefasciatus* | 15461 | *Canis lupus* |
| REYN052018F05 | *Culex quinquefasciatus* | 13455 | *Canis lupus* |
| REYN052018F06 | *Culex quinquefasciatus* | 103 | *Canis lupus* |
| REYN052018F07 | *Culex quinquefasciatus* | 148 | *Canis lupus* |
| REYN052018F09 | *Culex quinquefasciatus* | 134 | *Canis lupus* |
| REYN052018F11 | *Culex quinquefasciatus* | 11617 | *Canis lupus* |
| REYN052018G01 | *Culex quinquefasciatus* | 19765 | *Canis lupus* |
| REYN052018G01 | *Culex quinquefasciatus* | 761 | *Felis silvestris silvestris* |
| REYN052018G04 | *Culex quinquefasciatus* | 24974 | *Canis lupus* |
| REYN052018G04 | *Culex quinquefasciatus* | 6042 | *Columbina inca* |
| REYN052018G05 | *Culex quinquefasciatus* | 29528 | *Canis lupus* |
| REYN052018G07 | *Culex quinquefasciatus* | 101 | *Canis lupus* |
| REYN052018G08 | *Culex quinquefasciatus* | 248 | *Canis lupus* |
| REYN052018G09 | *Culex quinquefasciatus* | 143 | *Canis lupus* |
| REYN052018G10 | *Culex quinquefasciatus* | 23681 | *Canis lupus* |
| REYN052018G11 | *Culex quinquefasciatus* | 26078 | *Columbina inca* |
| REYN052018G12 | *Culex quinquefasciatus* | 119 | *Canis lupus* |
| REYN052018H01 | *Culex quinquefasciatus* | 10222 | *Canis lupus* |
| REYN052018H02 | *Culex quinquefasciatus* | 18139 | *Meleagris gallopavo* |
| REYN052018H02 | *Culex quinquefasciatus* | 105 | *Canis lupus* |
| REYN052018H03 | *Culex quinquefasciatus* | 129 | *Canis lupus* |
| REYN052018H05 | *Culex quinquefasciatus* | 19606 | *Canis lupus* |
| REYN052018H05 | *Culex quinquefasciatus* | 1734 | *Meleagris gallopavo* |
| REYN052018H06 | *Culex quinquefasciatus* | 22361 | *Meleagris gallopavo* |
| REYN052018H06 | *Culex quinquefasciatus* | 137 | *Canis lupus* |
| REYN052018H07 | *Culex quinquefasciatus* | 19311 | *Gallus gallus* |
| REYN052018H09 | *Culex quinquefasciatus* | 23242 | *Zenaida macroura* |
| REYN052018H09 | *Culex quinquefasciatus* | 125 | *Canis lupus* |
| REYN052018H11 | *Culex quinquefasciatus* | 17969 | *Canis lupus* |
| REYN062018A01 | *Culex quinquefasciatus* | 18115 | *Canis lupus* |
| REYN062018A02 | *Culex quinquefasciatus* | 11215 | *Canis lupus* |
| REYN062018A03 | *Culex quinquefasciatus* | 14036 | *Passer domesticus* |
| REYN062018A03 | *Culex quinquefasciatus* | 11007 | *Canis lupus* |
| REYN062018A04 | *Culex quinquefasciatus* | 14803 | *Canis lupus* |
| REYN062018A05 | *Culex quinquefasciatus* | 36207 | *Canis lupus* |
| REYN062018A06 | *Culex quinquefasciatus* | 14751 | *Canis lupus* |
| REYN062018A07 | *Culex quinquefasciatus* | 29767 | *Passer domesticus* |
| REYN062018A08 | *Culex quinquefasciatus* | 33583 | *Canis lupus* |
| REYN062018A09 | *Culex quinquefasciatus* | 18740 | *Canis lupus* |
| REYN062018A10 | *Culex quinquefasciatus* | 30307 | *Canis lupus* |
| REYN062018A11 | *Culex quinquefasciatus* | 30770 | *Canis lupus* |
| REYN062018A12 | *Culex quinquefasciatus* | 24213 | *Canis lupus* |
| REYN062018B01 | *Culex quinquefasciatus* | 21786 | *Canis lupus* |
| REYN062018B01 | *Culex quinquefasciatus* | 9217 | *Passer domesticus* |
| REYN062018B02 | *Culex quinquefasciatus* | 30461 | *Canis lupus* |
| REYN062018B03 | *Culex quinquefasciatus* | 18512 | *Canis lupus* |
| REYN062018B04 | *Culex quinquefasciatus* | 34042 | *Passer domesticus* |
| REYN062018B05 | *Culex quinquefasciatus* | 35433 | *Canis lupus* |
| REYN062018B06 | *Culex quinquefasciatus* | 24394 | *Canis lupus* |
| REYN062018B07 | *Culex quinquefasciatus* | 34977 | *Canis lupus* |
| REYN062018B08 | *Culex quinquefasciatus* | 26452 | *Canis lupus* |
| REYN062018B09 | *Culex quinquefasciatus* | 37521 | *Canis lupus* |
| REYN062018B10 | *Culex quinquefasciatus* | 29322 | *Canis lupus* |
| REYN062018B11 | *Culex quinquefasciatus* | 2563 | *Canis lupus* |
| REYN062018B12 | *Culex quinquefasciatus* | 23740 | *Canis lupus* |
| REYN062018C02 | *Culex quinquefasciatus* | 20696 | *Gallus gallus* |
| REYN062018C02 | *Culex quinquefasciatus* | 168 | *Canis lupus* |
| REYN062018C03 | *Culex quinquefasciatus* | 23282 | *Canis lupus* |
| REYN062018C04 | *Culex quinquefasciatus* | 25285 | *Gallus gallus* |
| REYN062018C05 | *Culex quinquefasciatus* | 15697 | *Canis lupus* |
| REYN062018C06 | *Culex quinquefasciatus* | 18885 | *Canis lupus* |
| REYN062018C07 | *Culex quinquefasciatus* | 20762 | *Canis lupus* |
| REYN062018C07 | *Culex quinquefasciatus* | 508 | *Butorides striata* |
| REYN062018C08 | *Culex quinquefasciatus* | 27740 | *Canis lupus* |
| REYN062018C09 | *Culex quinquefasciatus* | 27941 | *Canis lupus* |
| REYN062018C10 | *Culex quinquefasciatus* | 37790 | *Zenaida macroura* |
| REYN062018C10 | *Culex quinquefasciatus* | 107 | *Canis lupus* |
| REYN062018C11 | *Culex quinquefasciatus* | 28510 | *Canis lupus* |
| REYN062018C12 | *Culex quinquefasciatus* | 9812 | *Canis lupus* |
| REYN062018D01 | *Culex quinquefasciatus* | 27381 | *Canis lupus* |
| REYN062018D02 | *Culex quinquefasciatus* | 32139 | *Canis lupus* |
| REYN062018D03 | *Culex quinquefasciatus* | 19052 | *Thryomanes bewickii* |
| REYN062018D04 | *Culex quinquefasciatus* | 29936 | *Canis lupus* |
| REYN062018D05 | *Culex quinquefasciatus* | 20871 | *Canis lupus* |
| REYN062018D06 | *Culex quinquefasciatus* | 20024 | *Canis lupus* |
| REYN062018D06 | *Culex quinquefasciatus* | 383 | *Thryomanes bewickii* |
| REYN062018D07 | *Culex quinquefasciatus* | 27805 | *Canis lupus* |
| REYN062018D07 | *Culex quinquefasciatus* | 140 | *Didelphis virginiana* |
| REYN062018D08 | *Culex quinquefasciatus* | 27305 | *Canis lupus* |
| REYN062018D08 | *Culex quinquefasciatus* | 341 | *Equus ferus caballus* |
| REYN062018D09 | *Culex quinquefasciatus* | 250 | *Didelphis virginiana* |
| REYN062018D10 | *Culex quinquefasciatus* | 561 | *Equus ferus caballus* |
| REYN062018D10 | *Culex quinquefasciatus* | 101 | *Canis lupus* |
| REYN062018D11 | *Culex quinquefasciatus* | 8204 | *Thryomanes bewickii* |
| REYN062018D11 | *Culex quinquefasciatus* | 666 | *Felis silvestris silvestris* |
| REYN062018D12 | *Culex quinquefasciatus* | 14639 | *Canis lupus* |
| REYN062018E01 | *Culex quinquefasciatus* | 212 | *Canis lupus* |
| REYN062018E02 | *Culex quinquefasciatus* | 17434 | *Canis lupus* |
| REYN062018E03 | *Culex quinquefasciatus* | 38305 | *Canis lupus* |
| REYN062018E04 | *Culex quinquefasciatus* | 28015 | *Canis lupus* |
| REYN062018E05 | *Culex quinquefasciatus* | 30893 | *Canis lupus* |
| REYN062018E06 | *Culex quinquefasciatus* | 22615 | *Canis lupus* |
| REYN062018E07 | *Culex quinquefasciatus* | 44749 | *Canis lupus* |
| REYN062018E08 | *Culex quinquefasciatus* | 23527 | *Canis lupus* |
| REYN062018E09 | *Culex quinquefasciatus* | 25498 | *Canis lupus* |
| REYN062018E10 | *Culex quinquefasciatus* | 36306 | *Canis lupus* |
| REYN062018E11 | *Culex quinquefasciatus* | 32627 | *Canis lupus* |
| REYN062018E12 | *Culex quinquefasciatus* | 8385 | *Didelphis sp.* |
|  |  |  |  |
| REYN062018E12 | *Culex quinquefasciatus* | 272 | *Didelphis aurita* |
| REYN062018F01 | *Culex quinquefasciatus* | 104 | *Gallus gallus* |
| REYN062018F02 | *Culex quinquefasciatus* | 25307 | *Canis lupus* |
| REYN062018F03 | *Culex quinquefasciatus* | 28980 | *Canis lupus* |
| REYN062018F04 | *Culex quinquefasciatus* | 19808 | *Canis lupus* |
| REYN062018F05 | *Culex quinquefasciatus* | 21560 | *Canis lupus* |
| REYN062018F05 | *Culex quinquefasciatus* | 14231 | *Passer domesticus* |
| REYN062018F06 | *Culex quinquefasciatus* | 23140 | *Gallus gallus* |
| REYN062018F07 | *Culex quinquefasciatus* | 35729 | *Canis lupus* |
| REYN062018F08 | *Culex quinquefasciatus* | 23679 | *Canis lupus* |
| REYN062018F08 | *Culex quinquefasciatus* | 470 | *Homo sapiens* |
| REYN062018F09 | *Culex quinquefasciatus* | 2901 | *Canis lupus* |
| REYN062018F10 | *Culex quinquefasciatus* | 35204 | *Passer domesticus* |
| REYN062018F11 | *Culex quinquefasciatus* | 332 | *Canis lupus* |
| REYN062018G01 | *Culex quinquefasciatus* | 15339 | *Columba livia* |
| REYN062018G02 | *Culex quinquefasciatus* | 8091 | *Zenaida macroura* |
| REYN062018G02 | *Culex quinquefasciatus* | 4425 | *Columba sp.* |
|  |  |  |  |
| REYN062018G03 | *Culex quinquefasciatus* | 30398 | *Didelphis virginiana* |
| REYN062018G04 | *Culex quinquefasciatus* | 32095 | *Passer domesticus* |
| REYN062018G05 | *Culex quinquefasciatus* | 37681 | *Canis lupus* |
| REYN062018G06 | *Culex quinquefasciatus* | 16829 | *Canis lupus* |
| REYN062018G07 | *Culex quinquefasciatus* | 25359 | *Canis lupus* |
| REYN062018G08 | *Culex quinquefasciatus* | 26600 | *Canis lupus* |
| REYN062018G09 | *Culex quinquefasciatus* | 9439 | *Felis sp.* |
| REYN062018G09 | *Culex quinquefasciatus* | 649 | *Columba livia* |
|  |  |  |  |
| REYN062018G10 | *Culex quinquefasciatus* | 28195 | *Canis lupus* |
| REYN062018G10 | *Culex quinquefasciatus* | 811 | *Columba livia* |
| REYN062018G11 | *Culex quinquefasciatus* | 20250 | *Canis lupus* |
| REYN062018G12 | *Culex quinquefasciatus* | 24501 | *Canis lupus* |
| REYN062018H01 | *Culex quinquefasciatus* | 30558 | *Equus ferus caballus* |
| REYN062018H02 | *Culex quinquefasciatus* | 31092 | *Columba livia* |
| REYN062018H03 | *Culex quinquefasciatus* | 31879 | *Canis lupus* |
| REYN062018H04 | *Culex quinquefasciatus* | 27919 | *Canis lupus* |
| REYN062018H05 | *Culex quinquefasciatus* | 22378 | *Columba livia* |
| REYN062018H05 | *Culex quinquefasciatus* | 9432 | *Canis lupus* |
| REYN062018H06 | *Culex quinquefasciatus* | 32660 | *Quiscalus nicaraguensis* |
| REYN062018H07 | *Culex quinquefasciatus* | 36992 | *Canis lupus* |
| REYN062018H08 | *Culex quinquefasciatus* | 39811 | *Canis lupus* |
| REYN062018H09 | *Culex quinquefasciatus* | 24260 | *Canis lupus* |
| REYN062018H09 | *Culex quinquefasciatus* | 979 | *Columba livia* |
| REYN062018H10 | *Culex quinquefasciatus* | 25394 | *Canis lupus* |
| REYN062018H10 | *Culex quinquefasciatus* | 581 | *Zenaida macroura* |
| REYN062018H11 | *Culex quinquefasciatus* | 26226 | *Zenaida macroura* |
| REYN06A2018A01 | *Culex quinquefasciatus* | 15042 | *Canis lupus* |
| REYN06A2018A02 | *Culex quinquefasciatus* | 27058 | *Passer domesticus* |
| REYN06A2018A03 | *Culex quinquefasciatus* | 3617 | *Canis lupus* |
| REYN06A2018A04 | *Culex quinquefasciatus* | 33005 | *Canis lupus* |
| REYN06A2018A05 | *Culex quinquefasciatus* | 25099 | *Canis lupus* |
| REYN06A2018A06 | *Culex quinquefasciatus* | 23644 | *Canis lupus* |
| REYN06A2018A07 | *Culex quinquefasciatus* | 22823 | *Canis lupus* |
| REYN06A2018A10 | *Culex quinquefasciatus* | 30772 | *Canis lupus* |
| REYN06A2018A11 | *Culex quinquefasciatus* | 13636 | *Homo sapiens* |
| REYN06A2018A12 | *Culex quinquefasciatus* | 28617 | *Canis lupus* |
| REYN06A2018B01 | *Culex quinquefasciatus* | 29076 | *Canis lupus* |
| REYN06A2018B03 | *Culex quinquefasciatus* | 11340 | *Canis lupus* |
| REYN06A2018B04 | *Culex quinquefasciatus* | 189 | *Canis lupus* |
| REYN06A2018B05 | *Culex quinquefasciatus* | 156 | *Canis lupus* |
| REYN06A2018B07 | *Culex quinquefasciatus* | 10953 | *Canis lupus* |
| REYN06A2018B08 | *Culex quinquefasciatus* | 25822 | *Canis lupus* |
| REYN06A2018B09 | *Culex quinquefasciatus* | 31245 | *Canis lupus* |
| REYN06A2018B10 | *Culex quinquefasciatus* | 17363 | *Canis lupus* |
| REYN06A2018B12 | *Culex quinquefasciatus* | 16842 | *Canis lupus* |
| REYN06A2018C01 | *Culex quinquefasciatus* | 325 | *Canis lupus* |
| REYN06A2018C04 | *Culex quinquefasciatus* | 8079 | *Canis lupus* |
| REYN06A2018C05 | *Culex quinquefasciatus* | 25071 | *Canis lupus* |
| REYN06A2018C06 | *Culex quinquefasciatus* | 12625 | *Canis lupus* |
| REYN06A2018C07 | *Culex quinquefasciatus* | 10582 | *Canis lupus* |
| REYN06A2018C08 | *Culex quinquefasciatus* | 15539 | *Canis lupus* |
| REYN06A2018C09 | *Culex quinquefasciatus* | 7995 | *Canis lupus* |
| REYN06A2018C10 | *Culex quinquefasciatus* | 12688 | *Canis lupus* |
| REYN06A2018C11 | *Culex quinquefasciatus* | 25383 | *Canis lupus* |
| REYN06A2018C12 | *Culex quinquefasciatus* | 9708 | *Canis lupus* |
| REYN06A2018D01 | *Culex quinquefasciatus* | 20227 | *Canis lupus* |
| REYN06A2018D01 | *Culex quinquefasciatus* | 187 | *Gallus gallus* |
| REYN06A2018D02 | *Culex quinquefasciatus* | 22923 | *Canis lupus* |
| REYN06A2018D03 | *Culex quinquefasciatus* | 21329 | *Canis lupus* |
| REYN06A2018D04 | *Culex quinquefasciatus* | 24289 | *Canis lupus* |
| REYN06A2018D05 | *Culex quinquefasciatus* | 19231 | *Canis lupus* |
| REYN06A2018D06 | *Culex quinquefasciatus* | 10972 | *Canis lupus* |
| REYN06A2018D07 | *Culex quinquefasciatus* | 12930 | *Canis lupus* |
| REYN06A2018D08 | *Culex quinquefasciatus* | 22446 | *Canis lupus* |
| REYN06A2018D10 | *Culex quinquefasciatus* | 32104 | *Canis lupus* |
| REYN06A2018D11 | *Culex quinquefasciatus* | 27185 | *Canis lupus* |
| REYN06A2018D12 | *Culex quinquefasciatus* | 19910 | *Gallus gallus* |
| REYN06A2018E03 | *Culex quinquefasciatus* | 24447 | *Felis silvestris silvestris* |
| REYN06A2018E03 | *Culex quinquefasciatus* | 12152 | *Passer domesticus* |
| REYN06A2018E04 | *Culex quinquefasciatus* | 22438 | *Canis lupus* |
| REYN06A2018E05 | *Culex quinquefasciatus* | 12191 | *Canis lupus* |
| REYN06A2018E06 | *Culex quinquefasciatus* | 12116 | *Canis lupus* |
| REYN06A2018E08 | *Culex quinquefasciatus* | 21468 | *Canis lupus* |
| REYN06A2018E09 | *Culex quinquefasciatus* | 17179 | *Canis lupus* |
| REYN06A2018E09 | *Culex quinquefasciatus* | 4302 | *Homo sapiens* |
| REYN06A2018E10 | *Culex quinquefasciatus* | 33083 | *Canis lupus* |
| REYN06A2018E11 | *Culex quinquefasciatus* | 27302 | *Canis lupus* |
| REYN06A2018E12 | *Culex quinquefasciatus* | 28303 | *Canis lupus* |
| REYN06A2018F02 | *Culex quinquefasciatus* | 17544 | *Canis lupus* |
| REYN06A2018F03 | *Culex quinquefasciatus* | 220 | *Canis lupus* |
| REYN06A2018F04 | *Culex quinquefasciatus* | 20679 | *Canis lupus* |
| REYN06A2018F05 | *Culex quinquefasciatus* | 23156 | *Canis lupus* |
| REYN06A2018F07 | *Culex quinquefasciatus* | 35821 | *Canis lupus* |
| REYN06A2018F08 | *Culex quinquefasciatus* | 13501 | *Canis lupus* |
| REYN06A2018F10 | *Culex quinquefasciatus* | 21549 | *Canis lupus* |
| REYN06A2018F11 | *Culex quinquefasciatus* | 32411 | *Canis lupus* |
| REYN06A2018F12 | *Culex quinquefasciatus* | 12699 | *Canis lupus* |
| REYN06A2018G01 | *Culex quinquefasciatus* | 21895 | *Canis lupus* |
| REYN06A2018G02 | *Culex quinquefasciatus* | 13767 | *Canis lupus* |
| REYN06A2018G03 | *Culex quinquefasciatus* | 23166 | *Canis lupus* |
| REYN06A2018G04 | *Culex quinquefasciatus* | 36980 | *Passer domesticus* |
| REYN06A2018G05 | *Culex quinquefasciatus* | 18727 | *Canis lupus* |
| REYN06A2018G06 | *Culex quinquefasciatus* | 2726 | *Canis lupus* |
| REYN06A2018G07 | *Culex quinquefasciatus* | 23023 | *Canis lupus* |
| REYN06A2018G10 | *Culex quinquefasciatus* | 25246 | *Passer domesticus* |
| REYN06A2018G10 | *Culex quinquefasciatus* | 1039 | *Sus scrofa* |
| REYN06A2018G12 | *Culex quinquefasciatus* | 12540 | *Homo sapiens* |
|  |  |  |  |
| REYN06A2018H01 | *Culex quinquefasciatus* | 114 | *Canis lupus* |
| REYN06A2018H03 | *Culex quinquefasciatus* | 116 | *Canis lupus* |
| REYN06A2018H08 | *Culex quinquefasciatus* | 17341 | *Canis lupus* |
| REYN06A2018H11 | *Culex quinquefasciatus* | 9796 | *Homo sapiens* |
| REYN06A2018H11 | *Culex quinquefasciatus* | 6152 | *Canis lupus* |
