# Supplementary material for: Vertebrate-Aedes aegypti and Culex quinquefasciatus (Diptera)-arbovirus transmission networks: Non-human feeding revealed by meta-barcoding and next-generation sequencing
Source: PLoS Negl Trop Dis. 2020 Dec 31;14(12):e0008867. doi: 10.1371/journal.pntd.0008867 (PMC7806141; doi:10.1371/journal.pntd.0008867)
Supplement: S4 Table — (DOCX) [file pntd.0008867.s004.docx]

**S4_Table**: Host population densities documented by survey in the study area of Pedro J. Méndez neighbourhood, Reynosa, Northern Mexico.

| Common name | Latin name | Number observed |
| --- | --- | --- |
| Sparrow | *Passer domesticus* | 101 |
| Dove | *Columbina inca* | 84 |
| Collared dove | *Streptopelia decaocto* | 24 |
| Mourning dove | *Zenaida macroura* | 4 |
| Thick-billed kingbird | *Tyrannus crassirostris* | 1 |
| Curve-billed thrasher | *Toxostoma curvirostre* | 1 |
| White duck | *Anas platyrhynchus* | 2 |
| Muscovy duck | *Cairina moschata* | 1 |
| Mexican woodpecker | *Campephilus imperialis* | 1 |
| Broad-billed hummingbird | *Cynanthus latirostris* | 1 |
| Rock pigeon | *Columba livia* | 6 |
| Crow | *Corvus corax* | 66 |
| Chicken | *Gallus gallus* | 37 |
| White-tipped dove | *Leptotila verreauxi* | 1 |
| Black duck | *Anas rubripes* | 3 |
| Domestic goose | *Anser cygnoides* | 3 |
| Turkey | *Meleagris gallopavo* | 2 |
| Cat | *Felis silvestris silvestris* | 48 |
| Dog | *Canis lupus familiaris* | 65 |
| Pig | *Sus scrofa domesticus* | 3 |
| Red-eared turtle | *Trachemys scripta* | 3 |
| Horse | *Equus ferus caballus* | 7 |
| Donkey | *Equus africanus asinus* | 1 |
| Rabbit | *Oryctolagus cuniculus* | 2 |
| White-nosed coati | *Nasua narica* | 3 |
| Human | *Homo sapiens* | 79 |
