# Supplementary material for: Vertebrate-Aedes aegypti and Culex quinquefasciatus (Diptera)-arbovirus transmission networks: Non-human feeding revealed by meta-barcoding and next-generation sequencing
Source: PLoS Negl Trop Dis. 2020 Dec 31;14(12):e0008867. doi: 10.1371/journal.pntd.0008867 (PMC7806141; doi:10.1371/journal.pntd.0008867)
Supplement: Attachment — Submitted filename: Rebutal letter.docx [file pntd.0008867.s005.docx]

August 4th, 2020

**Pattamaporn Kittayapong**, Ph.D.
Associate Editor

PLOS Neglected Tropical Diseases (PLoS NTD)

**Rebecca Rico-Hesse**
Deputy Editor
PLOS Neglected Tropical Diseases

**Reference: PNTD-D-20-00295:**

"Vertebrate-*Aedes aegypti* and *Culex quinquefasciatus* (Diptera)-arbovirus transmission networks: Non-human feeding revealed by meta-barcoding and next-generation sequencing”.

Dear Drs. Kittayapong and Rico-Hesse

Thank you for your e-mail dated June 16th, 2020 regarding our above manuscript. We are delighted that the manuscript has been deemed acceptable for publication following suitable revision. I am including a revised version of the manuscript with this letter. It includes two versions of the manuscript, one with tracked changes using the Word function denoting where the text has been changed (uploaded as a "Revised Article with Changes Highlighted –RACH-" file); and the other is a clean version of the manuscript (uploaded as the “Article” file). In this revision, minor corrections and edits where made as indicated by reviewers. Other things such as order of original and new references included that we fixed and minor edits we did with track changes off so that they would not clutter up the RACH file.  I would like to summarize the comments of the two reviewers and our response (using **bold** letters) to him/her in the following sections.

**Reviewer # 1**

1) Would the authors mind to elaborate on the existence or necessity of an ethical committee's approval for the questionaries applied in the households of Reynosa?

**Author Response: Questionnaries were administered to 10 randomly selected households in the neighborhood, including households not involved in mosquito trappings. Basic informal questions such as the type and number of pets kept in the household were recorded as part of the animal survey. We have the following sentence inserted in the section of Methodology: "Ethical consideration: The present study was approved by the Bioethical Committee of the Faculty of Medicine at the Universidad Autónoma del Estado de México (Approval No. 011/2015) to JGEF".**

2) Line: 239: "...DENV was tested..." Did DENV detection also include a positive control, similarly to what was used for ZIKV and CHIVK?

**Author Response: Yes, all our RT-PCRs for virus testing included positive controls, including the DENV. As well as for ZIKV, CHIKV, and WNV. We have the following sentence inserted in the section of Methods:”DENV strains used as positive controls were provided by the University of Texas Medical Branch”.**

Line 367: "A total of 2,941 females Ae. aegypti were collected during the study."

1) Were any of these samples tested for a mosquito endogenous control gene, as to check for sample integrity?

**Author Response: For our blood meal analysis and virus testing we have not been using an endogenous control such as a housekeeping gene. An endogenous control would be a good idea but of course that increases the cost of the analysis. However, we routinely quantify nucleic acid recovered from the extraction on a spectrophotometer. In this way we at least verify the extraction process is working and we are recovering nucleic acid.  We have the following sentence inserted at lines 270-271: “For a subset of samples we quantified nucleic acid recovered using a spectrophotometer (Epoch, BioTek Instruments, Inc.).”**

2) After reading the methods section, I believe that all mosquitoes tested here did not contain a blood meal, correct? Can the authors add a few sentences on the rationale behind this selection approach in the discussion? This is a good opportunity to educate the readers on the importance of experimental design in the context of false positive results in vector competence studies due to undigested blood meal.

**Author Response: Yes, the reviewer is correct. As it is mentioned in Lines 139-141 “Female mosquitoes were further classified into different feeding stages and only engorged mosquitoes of Sella scale 2 (freshly fed) and 3 (late stage fed) were processed for the blood meal analysis” All other mosquitoes tested for the viruses did not containg a blood meal. Thus, the pooled mosquitoes would not have contained blooded individuals since those were saved for the blood meal analysis.**

**We have the following sentence inserted in the section of Discussion:“Pooled mosquitoes would not have contained blooded individuals to avoid false positive results when modeling the VC due to undigested blood meal.**

Lines 379-380: "According to the findings of Rodríguez-Pérez et al.34, six people were IgM-positive and seroconverted from a total of 77 examined people, therefore the Incidence/SH =6/(77-6)."

3) Is it correct to assume that this study was conducted at the same period in which the referenced work was also conducted?  If so, please state.

**Author Response: The reviewer is correct. The prevalence of dengue virus infection should be varying with time. We assumed that it is a constant due to the absence of such data. Similarly, the ratio of the number of new cases to the number of humans is also assumed to be constant. We assume this estimated incidence in the neighborhood Nuevo Amanecer can be extrapolated to the neighborhood (Pedro J. Mendez) of the study area as they are within the radius of the city of Reynosa with similar ecology and infraestructura conditions; There are also studies where vectorial capacity were calculated based on data collected elsewhere (PLoS Negl Trop Dis 2013;7(3):e2013. doi: 10.1371/journal.pntd.0002013. Epub 2013 Mar 7). The estimations would be more accurate if dengue prevalence/incidence data were available for the Pedro J. Mendez neighborhood, during the time this study was conducted, which it was not the case.**

**We have the following sentence inserted: “The prevalence of dengue virus infection should be varying with time. We assumed that it is a constant due to the absence of such data. Similarly, the ratio of the number of new cases to the number of humans is also assumed to be constant.”**

Lines 392-394: "Using the equation of vectorial capacity, we estimated VC of Ae. aegypti for DENV in Reynosa which ranged from 112 to 172 from temperatures varing between 22 and 30 ºC, respectively."

4) The word "varing" is misspelled. If chosen to keep the mathematical component of the paper, would the authors please include a plot for the vectorial capacity as a function of temperature given the parameters utilized in this study?

**Author Response: Thank you. The word “varing” was corrected, it is now spelled as ranging. A plot (Figure 4) for the VC as a function of temperatura was included: The relationship between temperature and vectorial capacity of *Aedes aegypti*.**

5) As for figures 2 and 3: I find this representation a bit hard to read given the color scheme selected and the amount of groups present. With that being said, I wonder if the authors would be willing to try a circular packing approach to display their data. It would allow to form big categories (circles) that are predominant, such as "dogs" containing inner circles of variable sizes (representing variable percentages) in which the mixed feeding behavior would be represented. The R code for such representation can be found at: [https://www.r-graph-gallery.com/circle-packing.html](https://www.r-graph-gallery.com/circle-packing.html) under the "several levels" category.

**Author Response:** **We thank the reviewer for this suggestion. We made several attempts to use the suggested circular R-package but it was not successful as we kept running into some errors with the codes. And we feel the bipartite network may not give a better representation either. We have however revised the figures and present them in a more suitable form using the Lollipop chart in R.**

Lines 427-431: "Although, chicken was the second most fed on host by proportion, forage ratio analysis revealed dog, Virginia opossum, and turkey as the most over-utilized hosts. The domestic dog was over-utilized by Cx. quinquefasciatus even more so than Ae. aegypti, with a 20-fold (79.2%) increase over the second most abundant host, chicken (3.7%)."

1) Could the authors please comment on the potential for the FR analyses to be biased, based on the sampling approach utilized? For instance, in general, birds are known to be more active early during sunrise or sunset, hence, an analyses conducted at around noon would potentially underestimate the abundance in a given area. I understand that it is hard to fully capture the species abundance in a given area, and this is totally acceptable in my point of view, however I few like a few sentences stating this issue should be present here in the discussion.

**Author Response: As reviewer suggested, we have inserted the following: “Our forage ratio analysis was based on a survey of animals present in these communities.  Given that we were attempting to sample a combination of wild and domestic animals, we used a combination of a ‘census’, by counting all animals observed during point counts as well as a ‘survey’, in which a subsample of the animals present was estimated.  We asked the homeowners for the number of animals in each household.  The forage ratios are based on the relative abundance of these vertebrates which would be difficult to compare wild animals (such as birds) to domestic animals (such as dogs) given the difference in survey methodologies.  The interpretation of the high forage ratio on dogs is difficult to interpret for this reason.  Prior published studies report that *Culex quinquefasciatus* are ornithophilic^45^, so the difference compared to the current study could be due to host availability”.**

**We also appreciate and understand the reviewer's, and indeed the animal survey was done between the early hours of 8-10 am in the study area. And like rightly pointed out, only an estimate of species abundant can be provided as it is difficult to fully capture all. We have the following sentences inserted in the sections of Methods and Discussion to address also this.**

**Line 255: ".... between 08:00 and 10:00 hrs. when the avian population will still be relatively more active.”**

**Lines 467-468: " Estimating the relative density of wild and domestic animals is difficult, and although our study found that chicken...."**

Lines 467-468: "A plausible explanation for the low anthropophily observed in Ae. aegypti could be the relatively low human density compared to dogs."

2) Can the authors expand the discussion section to include a paragraph describing the sociodemographic as well as epidemiological status of Reynosa for the arboviruses here assayed? It would give the readers, not familiar with the region, a better understanding of the results here presented.

I would like to expand on the importance of such information, by giving an example based on a previous work I was involved with.

In a certain low-income area with high incidence of dengue in the population, we observed that the deployment of our vector control strategy did not have a major impact on case numbers. After a careful sociodemographic evaluation, it became clear to us that the issue was not associated with the vector control strategy by itself, but with how the population dynamics work in that area.

Given the low-income aspect of the area and the lack of business to provide jobs for its inhabitants, a vast majority of the individuals in that area would spend around >80% of its time, outside the area, working, hence acquiring dengue outside the region in which we deployed our vector control strategy. As such, the result here present makes me wonder if what we observed in our study would also be a potential explanation for the high feeding rate in dogs and other hosts rather than humans. For instance one is to expect dogs to spend all day in a certain area whereas the same would most likely not be the case for the pet owners/humans, hence the chances of a mosquito encountering a host other than humans to blood feed would be higher.

**Author Response: The description of the study area, now to be included in the methodology has addressed the sociodemographics requested. The following have been included in Lines 587-589 to address the epidemiological status requested:  ".... Although an earlier study in a similar Reynosa neighborhood isolated CHIKV from patients with dengue-like symptoms^56^ and co-circulation of DENV1-3 and West Nile virus^57^, all …."**

Lines 522-525: "For example, ivermectin has been reported to be a safe, short-term insecticide useful for malaria control in areas where mosquitoes are exophagic or exophilic50. In some cases, mosquito populations were reduced up to 80% after feeding on ivermectin treated individuals51."

3) I would avoid mixing studies on parasites with the idea of arboviruses control. If the authors want to support their argument, then please use references in the context of the same vector/virus system. For instance DOI:10.1603/me11164 is a work using the same drug in Ae. aegypti, rather than Anopheles mosquitoes.

**Author Response: We have kept the references of the effect of ivermectin on Anopheles and the following sentence inserted in the section of Discussion: “We also support our argument that anti-parasitic drugs can have an effect on vector populations in the context of the same vector/virus system. For instance, Deus et al. (2012)^55^ found that ivermectin induces *Ae. aegypti* adult mortality and decreases the hatch rate of their eggs.”**

Lines 526-527: "Diversion of vector bites to incompetent arbovirus host maybe responsible for the low transmission rates observed in the study area."

4) I find this sentence confusing and rather difficult to understand. Would the authors mind to rephrase it?

**Author Response: The original sentence was rephrased as to: “Diversion of vector bites to non-competent hosts reduces viral amplification and reduces potentially infectious bites to humans by dengue vectors, thus could be used as a control tool to limit arbovirus transmission.”**

Lines 541-543: "Although dogs may be unable to achieve the infectious viremia of ZIKV, seroconversion and neutralizing antibody production increases the opportunity of detecting low level arbovirus transmission."

5) I would like to ask the authors to please briefly expand on such argument. For instance, before proposing such alternative approach, is there any data in the literature to predict such strategy as a reliable source for Flavivirus surveillance, in the context of antibody specificity and cross-reactivity?

**Author Response: We have the following sentence inserted in Lines 603-609: “Several studies have suggested dogs can be a sentinel for WNV^11^ and a study is underway in Mexico investigating dog seroconversion to flaviviruses including ZIKV, DENV, and WNV (unpublished data).. Thus, although….”**

Lines 545-549: "respectively). However, if h were 100% (i.e. all Ae. aegypti biting humans; 97% of Ae. aegypti feeding on humans was documented in Thai and Puerto Rico)39, VC would increase 2.5 times (ranging from 287 to 437, respectively). Hence, if the proportion of Ae. aegypti bloodmeals on humans were even higher than that reported here, the transmission of dengue viruses in this area would likely be much higher."

6) I would recommend removing this hypothetical scenario based on feeding rates observed in other regions and just focus on the results collected by the authors. With that being said, I am not particularly enthusiastic about mathematical models based on data collected elsewhere and I am not convinced that the model here presented adds any relevant information to the work, but rather interfere with the reader's focus on the novelty aspect of this work, which is the bar coding/NGS approach to identify host feeding behavior. My recommendations would for the authors would be to:

A) Completely remove the vectorial capacity component of this manuscript, as it heavily relies on data from other regions/mosquito population genetics which might under or overestimate the results here obtained.

B) Leave the mathematical model, but include a paragraph expanding on the limitations of the model here used in the context of data availability/collection.

**Author Response: There are studies where CV were calculated based on data collected elsewhere; for example, the authors presented the impact of entomopathogenic fungus on CV of *Aedes aegypti* (PLoS Negl Trop Dis 2013;7(3):e2013. doi: 10.1371/journal.pntd.0002013. Epub 2013 Mar 7). We have adopted option B as suggested by reviewer and a paragraph in the section of Discussion was inserted as reviewer have also suggested: “The vector competence is the product of several factors, and should be varying with time. For example, the factor I_M_(t)/N__M_(t), which represents the prevalence of dengue virus infection should be varying with time. We assumed that it is a constant due to the absence of such data. Similarly, The ratio of the number of new cases to the number of humans is also assumed to be constant. Some parameters that are unavailable were derived from literature, such as human biting rate, a, and the ratio of the number of mosquitoes to the number of humans are also estimated assuming that there are four people in one household. The survival rate varying with time is also from literature. The estimations would be more accurate with more data available.”**

Line 83: "found globally in world..."

**Author Response: This was changed to worlwide**

Line 94-95: "Various techniques that have been employed for the analysis of mosquito blood meals such as ELISA or precipitin tests6, 7, and PCR-based approaches8."

**Author Response: This was changed to: Various techniques have been employed for the analysis of mosquito blood meals such as ELISA or precipitin tests^6, 7^, and PCR-based approaches^8^.**

Line 121: "Mosquito". **Please correct it to Mosquitoes**

**Author Response: This was done.**

Line 128: "Ovitraps (AGO) traps (Springstar) that was checked weekly15" Replace was by were

**Author Response: This was done.**

Line 135: "Mosquito identifications was based"

**Author Response: This was changed to: Mosquito morphology identification was conducted using standard keys.**

Line 157: "0.5 mMEDTA pH"

**Author Response: This was done.**

I believe that the use of NGS technology and the blood feeding behavior results obtained through such technique for two key arboviral species is already sufficient to qualify this work for publication. The addition of a mathematical model for vectorial capacity, mainly based on parameters estimated by others using different mosquito populations from other regions, does not add any value to the manuscript, but rather remove some of the focus on the results obtained. Given the lack of a proper expansion on the implications of such model in all sections of the paper, particularly in the discussion section, I would recommend for the complete removal of it.

**Author Response: A plot (Fig. 4) for the VC as a function of temperatura was included: The relationship between temperature and vectorial capacity of *Aedes aegypti*. We have also included a paragraph mentioning the limitations of the model here used for vectorial capacity of *Aedes aegypti*.**

**Reviewer #2**

Not sure if IRB needed for household interviews if done.

**Author response: Questionnaries were administered to 10 randomly selected households in the neighborhood, including households not involved in mosquito trappings. Basic informal questions such as the type and number of pets kept in the household were recorded as part of the animal survey.**

**The present study is not defined as human subjects research, thus it could be exempt from IRB review and approval; however, to answer to the reviewer concerns, we have the following sentence inserted in the section of Methodology: "Ethical consideration: The present study was approved by the Bioethical Committee of the Faculty of Medicine at the Universidad Autónoma del Estado de México (Approval No. 011/2015) to JGEF".**

Figure 3 and perhaps 2 could be much improved (the blood meal analyses) by visualizing them as bipartite network mapping with visualization of species  instead of that especially confusing pie chart in Figure 3. This should be improved, in my opinion.

**Author Response: Figures 2 and 3 were re-formated as requested by reviewer.**

The limitations of the analyses could be improved.

**We have included a paragraph mentioning the limitations of the model here used for vectorial capacity of *Aedes aegypti*.**

Line 289 'sampling' should not be italicized

**Author Response: Actually, the word italicized is *where*. Now, this is fine.**

The neighborhood is poorly described. Is this a rural or urban area? How many people live in the neighborhood sampled? What is the typical household constructed of? What is the general housing and yard infrastructure in the sample area. How large is this neighborhood (how many households in total) and how were these 16 households chosen? How representative was the household sampling of the neighborhood of study? How did you determine they were representative of the range of habitats for the mosquito vectors. What is the relative spatial distribution of these households and does this impact patterns of blood meal feeding patterns and infection even? How permeable are households to mosquitoes? Do inhabitants spend a lot of time outside?

Please provide a description of household and yards (type, etc).

**Author Response: We appreciate the reviewer's comments and have now provided an additional description of the neighborhood in the section of Methodology:**

**“The study area was residential and the objective of the study is to understand vector behavior in this residential area. The household selection was random and largely dependent on permission granted by house owners as well as availability to allow entrance on their properties to check traps and retrieved mosquitos. Mosquito collection was done mostly outdoors, in the yard of these properties (3 out of the 4 traps viz: AGO and BG traps, and the resting boxes were all outdoors traps, with only the backpack aspiration, done inside). Also, the nature of the study area, where all of the houses shared fences, provided opportunity to sample mosquitoes within a 50-meter radius of each outdoor trap, as this is within the flight range of mosquitoes. Furthermore, with a sampling duration of 5 months, we have a strong reason to believe there is enough representation of the mosquito population in this area.  We did not attempt to quantify the behavior of the household occupants and how much time was spent inside versus outside. However, anecdotally we observed a significant proportion of their activities, including cooking, grilling, and evening family gatherings, were done outside. Permeability of houses to mosquitoes as well was not measured. Given mosquitoes can have a flight range of up to 100 m post-feeding^18^ and species of *Aedes* and *Culex* have 2-5 km flight range^19^, we do not think the spatial distribution of the households might have significantly impacted the bloodmeal feeding pattern observed".**

**The following sentence was also added: “The neighborhood is a semi-urban characterized by infrastructure deficit. Intrastreet roads are unpaved, and houses are mostly incomplete or unplastered. Waste management practices is poor and streets are often flooded after rainfall. The population of the area is about 7,031 people over a *ca.* 6,016 square/meter comprising an estimated 1,750 households”.**

I might have missed this but were bugs pooled at the household level  or in general at community level when tested for viruses?

**Author Response: Mosquitoes were pooled at the household level by trap-type and date of collection.**

Since you saw a relatively low number of mosquito feeding on humans (esp. for quinquefasciatus), did you do any behavioral studies evaluating how often people are resting or active outside around the households of study, or do people spend most of their time indoors in these neighborhoods?

This has been responded to in one of the earlier responses sent. And it was discussed further in Lines 490-496 in the manuscript

**Author Response: This has been responded to in one of the earlier responses. And it was discussed further in Lines 144-161 in the manuscript.**

Figure 3 and perhaps 2 should be improved visually (some type of bipartite network)

**Author Response: Figures 2 and 3 were improved as suggested.**

We certify that this material has not and will not be offered elsewhere for possible publication, as long as it is under consideration by the *PLOS Neglected Tropical Diseases*. All authors of this paper agreed to allow me to submit revisions on their behalf in the original cover letter. We have returned the signed page charge agreement for this manuscript previously. We hope that the revisions that we have made in this manuscript will render it suitable for final publication in the *PLOS Neglected Tropical Diseases*.

Thank you in advance for taking the time to consider this manuscript.

Sincerely yours,

**Dr.**

**Corresponding and senior author**
