# Supplementary material for: Vertebrate-Aedes aegypti and Culex quinquefasciatus (Diptera)-arbovirus transmission networks: Non-human feeding revealed by meta-barcoding and next-generation sequencing
Source: PLoS Negl Trop Dis. 2020 Dec 31;14(12):e0008867. doi: 10.1371/journal.pntd.0008867 (PMC7806141; doi:10.1371/journal.pntd.0008867)
Supplement: Attachment — Submitted filename: Rebuttal letter.docx [file pntd.0008867.s006.docx]

September 17th, 2020

**Pattamaporn Kittayapong**, Ph.D.
Associate Editor

PLOS Neglected Tropical Diseases (PLoS NTD)

**Rebecca Rico-Hesse**
Deputy Editor
PLOS Neglected Tropical Diseases

**Reference: PNTD-D-20-00295:**

"Vertebrate-*Aedes aegypti* and *Culex quinquefasciatus* (Diptera)-arbovirus transmission networks: Non-human feeding revealed by meta-barcoding and next-generation sequencing”.

Dear Drs. Kittayapong and Rico-Hesse

Thank you for your e-mail dated September 10th, 2020 regarding our above manuscript. We are delighted that the manuscript has been deemed acceptable for publication following suitable revision. I am including a revised version of the manuscript with this letter. It includes two versions of the manuscript, one with tracked changes using the Word function denoting where the text has been changed (uploaded as a "Revised Article with Changes Highlighted –RACH-" file); and the other is a clean version of the manuscript (uploaded as the “Article” file). In this revision, minor corrections and edits where made as indicated by reviewers. I would like to summarize the comments of the two reviewers and our response (using **bold** letters) to him/her in the following sections.

**Reviewer # 1**

The legend for figure 4 needs more information. Include a reference for the papers in which the models were based, as they are currently buried in the methods section. Specify that the model is for the mosquito population in general, instead of an overall model of vectorial capacity for *Aedes aegypti*.

**Author Response: This was done. Legend of Fig. 4 now reads: The relationship between temperature and vectorial capacity of *Aedes aegypti*. Extrinsic incubation period, vector competence, survival rate, and vectorial capacity were calculated using models as reported elsewhere^31-34^. It should be clarify that the vectorial capacity model used is for the mosquito population in general, instead of an overall model of vectorial capacity for *Aedes aegypti*.**

Please, revise the manuscript one more time, as there are many typos and misspelled words.

**Author Response: This was done. We have included edits in the manuscript to improve the English in a few spots.**

**Reviewer #2**

Table 2 and 3- Please rank species by forage ratio or % of host fed level in order from highest to lowest, so it is easier to interpret the data. Also, Figures 2 and 3 would be easier to read using a bipartite network where blood meal-mosquito associations can be evaluated and visualized in a more elegant way that is clear for the reader to interpret.

**Author Response: Tables 2 and 3 were re-formated as requested by reviewer. We thank and appreciate the reviewer's suggestion of using a bipartite network for Figures 2 and 3, we would however like to maintain the figures as they currently are.  We have changed these figures from what was initially presented on the suggestions of the reviewer and we strongly believe the information we are trying to communicate is clear enough with this current form. We do not believe the bipartite network has any significant advantage over the current one.**

**Generic reviewer comments of “for the most part” or “more or less”, “not completely”, “nearly completely”, “for the most part adequately addressed” are hard to deal with. Thus, we could not address those comments.**

**We also made a correction in line 592. As the PLOS data access policy requires that all data be either published with the manuscript or made available in a publicly accessible database, we have removed the following sentence: “…and a study is underway in Mexico investigating dog seroconversion to flaviviruses including ZIKV, DENV, and WNV (unpublished data)”. Therefore, we have the following sentence corrected in the section of Discussion: “Thus, although dogs may be unable to achieve the infectious viremia of arboviruses, seroconversion and neutralizing antibody production increases the opportunity of detecting low level arbovirus transmission”.**

We certify that this material has not and will not be offered elsewhere for possible publication, as long as it is under consideration by the *PLOS Neglected Tropical Diseases*. All authors of this paper agreed to allow me to submit revisions on their behalf in the original cover letter. We have returned the signed page charge agreement for this manuscript previously. We hope that the revisions that we have made in this manuscript will render it suitable for final publication in the *PLOS Neglected Tropical Diseases*.

Thank you in advance for taking the time to consider this manuscript.

Sincerely yours,

**Dr.**

**Corresponding and senior author**
